# A Medical Aspect of War Wastage

**Published:** 1916-02-19

**Authors:** 


### THE SOLDIER'S HEART.

### A Medical Aspect of War Wastage.

The stress of war and the incidents of battle have an obvious association with the practice of surgery, and in this direction the medical profession has during recent events both rendered many services and learned many lessons. Medicine, with its greater complexities and uncertainties, makes a much less dramatic appeal, and necessarily holds a less prominent consideration, at least in the public estimate. Yet the war has brought opportunities to the physician, and it is well to know that these are being utilised in the interest both of the individual patient and of medical science. A recent discussion at the Royal Society of Medicine affords an illustration of this statement. For various reasons the last few years have witnessed much attention to diseases of the heart, and the subject more recently has necessarily been prominent in regard both to recruits and to soldiers. There have been some differences of opinion as to the interpretation of physical signs as bearing on the question of organic disease and on the capacity of men to undergo the fatigues and stresses of an active campaign. But the quarrel, we fancy, has been one rather of words than of substance. Rival experts may dispute about general propositions, but in dealing with an individual case it may be doubted whether they would be very far apart. Here, indeed, is the difficulty of detecting reality in the strife. In the individual instance the whole man is in evidence, not merely certain heart sounds, but on paper the latter monopolise attention, and no verbal description really completes the clinical picture. In the discussion at the Royal Society this particular dispute was not included. Interpretation of sounds, normal or abnormal, had no place, and the issue was confined to instances in which there exists a persisting tendency to unduly rapid cardiac action. The cause of this condition, and the best method of treating it—these were the topics for debate.

It appears that the "irritable heart of soldiers" has attracted the attention of the military medical experts on many occasions during the last half-century. Government committees have sat upon it, and medical scientists have investigated it—and yet it is with us still. At one time tight accoutrements were accused as its cause, at another time "setting-up drill." The drill and accoutrements were alike got rid of, but the irritable heart remains. A new attempt is now to be made to solve the mystery, and the sufferers are to be collected in a large hospital where all the modern methods of medical science may be applied to them in definitely organised fashion. Systematic observation conducted under exact conditions is to be utilised for the collection of hard facts, and out of these facts it is hoped to wrest the secret of the cause and cure of the irritable heart. The wise men profess their ignorance, and are content to await the results of the contemplated inquiry, but there are others who have been convinced in advance. Some of these point an accusing finger at tobacco, or at least at cigarettes, others cast doubts on the thyroid gland and urge *x*-rays as the certain cure, while yet again we hear of the inevitable efficacy of Nauheim baths "as administered in London"—a suggestion without which no discussion on heart disease can nowadays be regarded as complete.

The "irritable heart" manifests itself in various fashions. Its action may be unduly rapid or, though quick even on slight exertion, it may be normal during rest. Breathlessness, also, is easily provoked by bodily activity, and sometimes there is pain in the heart region. With these symptoms there may be more technical signs of heart disturbance, such as some increase in size, the presence of murmurs, and even dropsical swelling of the feet. In his general condition the patient is usually mentally depressed, takes a gloomy view of his condition and prospects, is conscious that he is not "fit," and sometimes describes himself as feeling "rotten." Indeed, this general state of unfitness and of exhaustion and depression is a commanding feature of the condition, and it therefore seems highly probable that the prejudicial agency, whatever it be, acts harmfully on the whole of the nervous system, and that the heart disturbance is only one manifestation of a widespread nervous inefficiency. Hence it is not surprising to find that the "irritable heart" displays itself in those who have been subject to prolonged or unusual mental stress, and that men who have suffered the strain of the trenches and have lost much sleep are among its victims. There are, however, other patients to whom no such explanation applies, and at least some of these have passed through an illness in which there have been febrile temperatures. According to modern views such temperatures usually mean the invasion of the body by microbes, which, by the toxins they produce, poison the blood and tissues. It would, therefore, look as if certain of these toxins could exercise a harmful effect on the muscular or nervous mechanisms of the heart, and one of the purposes of the contemplated investigation will be to settle this point. Another practical aspect of the same question is important from the patient's point of view. If, instead of being told he has a "weak heart," he can be assured that all his symptoms, including the palpitation and breathlessness, are due merely to nervous exhaustion, he is at once relieved from a great dread and much is done to facilitate his recovery. Towards the same end is a contribution from the treatment he will be ordered. So long as under the fear of a "weak heart" he is condemned to a long rest, followed by great restriction of his activities, he must needs feel anxiety about his condition, and must live the life of a chronic invalid. But when his "irritable heart" is explained to him as merely part of a general nervous exhaustion, and he is encouraged to a tonic *régime* including graduated exercises and enjoyable games in the open air, the future takes on another complexion, and the hopefulness thus infused is an important influence in the re-establishment of good health. Somehow or other all this does not sound particularly new, but a systematic investigation may put the whole question on a scientific basis and thus may render more confident the clinical distinction between an "irritable heart" and genuine heart disease. There is no doubt that patients are now and again condemned to a life of unnecessary restrictions on the ground of "heart weakness" when they would be all the better for duly supervised activities.

